# 

**Published:** 1881-01

**Authors:** 


PAGE

ORIGINAL COMMUNICATIONS—Recent Contributions to the Literature of Comparative Medicine. By Thomas E. Satterthwaite, M.D. ........ 1
Anæsthetics in Veterinary Practice. By W. H. Porter, M.D. ........ 12
Diseased Meat and its consequences upon our Health and Happiness. By Noah Cressy, M.D., V.S., Ph.D. ........ 15
Sterility of Mammalia under Confinement. By Arthur Erwin Brown ........ 28
The Sanitary Condition of New York City Stables By Allen S. Heath, M D. ........ 36
Enteritis. By G. H. Berns, D.V.S. ........ 38
EDITORIAL—The Prevention of Infectious Disease by Inoculation ........ 41
Experiments Regarding Anthrax ........ 43
State Help for Investigations into Contagious Diseases of Animals ........ 43
A National Cattle Commission ........ 44
Tuberculosis and Experiments on Murderers ........ 45
Sanitary Care of the Lower Animals ........ 46
Heavy Draught Breeding Horses—Veterinary Education in England ........ 47
The Journal of Comparative Medicine ........ 48
REVIEWS—Horses' Teeth. By W. H. Clarke ........ 49
Compend of Anatomy. By John B. Roberts, A.M., M.D ........ 50
The Beef Bonanza. By Gen. J. S. Brisbin, U. S. A. ........ 50
CASE DEPARTMENT—Œdema Glottidis following the Epizooty ........ 52
Chronic Diffuse Nephritis ........ 53
Acute Congestion of the Kidneys with Posterior Paralysis ........ 55
Scirrhous Cancer in a Cow ........ 56
Fibrinous Pericarditis in the Horse, with Purpura Hæmorrhagica ........ 57
Phthisis in a Monkey ........ 60
CORRESPONDENCE—Case of Meningitis in an Elephant. By Chas. A. Todd, M D. 61
Raids on the Central Park Menagerie. By W A. Conklin ........ 63
PROGRESS OF VETERINARY SCIENCE—The Standard Ration for Horses—Spurious Pregnancy in the Lower Animals—The Gastrodiscus Sonsinoii—Experiments in Horse Feeding—Cows Remaining in Heat—Malignant Pustule and Œdema of Anthrax—The Latest Theory Regarding the Abortion of Cows—Ricketty Eggs—Scurfy Legs in Fowls—Bumble-Foot—Fowl Cholera in the West—Dyspepsia in Pigs—Milk Sickness, or Trembles—The Embryo of the Tape-worm—The Effect of Alcohol on Pigs ........ 64
NEWS AND MISCELLANY—A Manual of Operative Veterinary Surgery—A Giant Horse—Veterinary Society in Australia—Regulating Veterinary Practice—A New Disease—Contagiousness of Epizootic Discharges—Dr. Charles D. House—Number of Horses in the World—Fasting Horses—Trichinosis in Spain—Suppressing Pleuro-Pneumonia—Horse Trade in Cincinnati—The Trade in Live Cattle—Contagious Diseases Among American Cattle—Parasites in Flies—Extinction of Pleuro-Pneumonia in Holland—The Increased Transportation of Cattle—Foot and Mouth Disease in England—Texas Cattle Fever not Epidemic in Texas—Transit of Cattle on Railway Cars—Appropriations for Experiments—Losses from Hog-Cholera—Fasting Frogs—A National Cattle Commission. 70

